# A novel multiplex approach for the comprehensive analysis of the Epstein-Barr virus-specific humoral immune response

**DOI:** 10.3389/fcimb.2026.1829807

**Published:** 2026-05-18

**Authors:** Carola Meindl, Ramona Weggel, Karolin Deichl, Kathrin Maria Fleischer, Paula Hofmeier, Mette Last, Julius Nückel, Elisa Planatscher, Lobna Ali Onsi, Anna Schütt, Uta Behrends, Josef Mautner

**Affiliations:** 1 Children’s Hospital, School of Medicine, Technische Universität München, Munich, Munich, Germany; 2 German Centre for Infection Research, Deutsches Zentrum für Infektionsforschung (DZIF), Partner Site Munich, Munich, Germany; 3 Institute of Virology, School of Medicine, Technische Universität München & Helmholtz Zentrum München, Munich, Germany; 4 Department of Microbiology and Immunology, German University in Cairo, Cairo, Egypt

**Keywords:** antigen array, diagnostics, Epstein-Barr virus, immunoglobulin, multiplex serology

## Abstract

**Background:**

The Epstein-Barr virus (EBV) is an oncogenic herpesvirus that establishes lifelong infections in more than 90% of the human population. EBV infection is typically diagnosed using serological assays, which also distinguish between acute and persistent infection. However, current diagnostic approaches rely on a limited set of viral proteins and may therefore fail to detect disease-specific antibody signatures associated with EBV-related cancers or autoimmune diseases. Comprehensive profiling of humoral responses against the complete EBV proteome could improve the sensitivity and specificity of serological diagnostics.

**Methods:**

We developed a serological multiplex assay covering all EBV proteins expressed in human cells, enabling measurement of virus-specific IgM, IgG, and IgA responses. The assay was evaluated using sera from healthy EBV carriers and EBV-negative controls. Near-infrared detection was employed to support quantitative analysis across a broad dynamic range. Performance was benchmarked against commercially available serological assays.

**Results:**

The multiplex assay demonstrated a broad dynamic range with linear quantification across several orders of magnitude. Comparative evaluation showed that the assay achieved sensitivity and specificity comparable to established commercial tests while enabling comprehensive antibody profiling against the full EBV proteome.

**Conclusion:**

This multiplex serological platform provides a robust approach for comprehensive characterization of EBV-specific humoral immune responses. By extending antibody profiling beyond the limited antigen panels used in routine diagnostics, the assay may facilitate the identification of novel biomarkers for EBV-associated cancers and autoimmune diseases. Future applications could contribute to improved diagnostic accuracy and support the development of targeted therapeutic strategies.

## Introduction

The oncogenic Epstein-Barr virus (EBV) establishes lifelong persistent infections in more than 90% of the human population (Longnecker et al., 2013; Damania et al., 2022). Although contained as asymptomatic infection in most virus carriers, some infected individuals develop EBV-associated malignancies that account for 1-2% of all human cancers (Farrell, 2019). Moreover, EBV infection has been associated with the development of some inflammatory and autoimmune disorders (Longnecker et al., 2013; Damania et al., 2022). Infection with EBV is usually diagnosed serologically, in the past by detecting heterophile antibodies, at present by measuring virus-specific antibodies using different detection systems such as immunofluorescence, chemiluminescence, enzyme immunoassays, multiplex flow immunoassays, and immunoblot (Niller and Bauer, 2017; Mautner and Middeldorp, 2025; Middeldorp, 2015). Diagnostic tests typically target a small set of viral antigens that usually include the viral capsid antigens and the Epstein-Barr nuclear antigen 1 (EBNA1), sometimes in combination with proteins of the early antigen (EA) complex. Combinations of IgM and IgG antibody responses against these antigens reliably detect seroconversion and facilitate discrimination between acute and persistent EBV infection (Niller and Bauer, 2017).

However, no consistent disease-specific serological profiles have been identified for other EBV-associated diseases (Coghill and Hildesheim, 2014; Fontes-Lemus et al., 2023; Sinha et al., 2022). In nasopharyngeal carcinoma, the EBV-associated malignancy most thoroughly investigated for serological biomarkers, elevated anti-VCA IgA and anti-EBNA1 IgA titers have been postulated as potential biomarkers for the detection of early, still highly curable NPC. However, although increased anti-VCA IgA and anti-EBNA1 IgA titers were shown to precede the manifestation of NPC sometimes for years, and anti-EBV IgA tests were able to improve early diagnostic rate and decrease NPC mortality, the positive predictive value in recently initiated secondary prevention programs in endemic disease areas was only about 4%, resulting in low compliance and screening efficiency (Lupo et al., 2023; Ji et al., 2019). Recently, antibodies against BNLF2b were identified as new candidate serological biomarker and the combination of EBNA1-IgA and BNLF2b-IgG was reported to improve NPC discrimination by 15.8% compared to the traditional anti-EBNA1 and anti-VCA IgA-based approach (Ma et al., 2024; Li et al., 2023). While encouraging, further validation through prospective studies is still needed. Likewise, patients with multiple sclerosis usually display heightened titers of antibodies against EBNA1 and VCA, and high anti-EBNA1 IgG responses are predictive of MS development (Bjornevik et al., 2023), but these antibody responses are unlikely to represent diagnostic or prognostic biomarkers in MS (Bose et al., 2023).

Given the limited clinical utility of currently available commercial tests in most EBV-associated diseases, different studies have assessed additional viral antigens as potential biomarkers of EBV infection and associated disorders such as EBNA2, the EBNA3 family of proteins, BZLF1, BLLF1 (gp350/220), and many more (Goswami et al., 2017)(and references therein). These studies revealed a remarkable broadness of the EBV-specific antibody response, but none of the candidate antigens was able to significantly improve diagnostic validity over standard serological tests.

More recent studies, therefore, sought to profile the antibody response against the entire EBV proteome consisting of more than 80 viral proteins. In contrast to earlier studies utilizing lysates of lytically EBV-infected cells with highly variable EBV protein levels, recent studies focused on recombinant EBV proteins and polypeptides as source of antigen produced chemically or in prokaryotic or eukaryotic expression systems (Goswami et al., 2017; Xu et al., 2015; Coghill et al., 2018; Zheng et al., 2011). Because proteins lack post-translational modifications when expressed in *E. coli* and human glycosylation structures when expressed in yeast (Hamilton and Zha, 2015), antibody responses were shown to vary depending on the source proteins (Emini et al., 1988). Moreover, donor-specific variations in the humoral immune response against residual components of the heterologous protein-producing cell preclude predefining cut-off values and, hence, clear discrimination between positive and negative responses. Besides, differences in standardization, dynamic range, and quantification were shown to impair reproducibility of serological tests when compared between laboratories (Liu et al., 2019).

To prevent distorted antigenic profiles and to establish positivity cut-offs for future standardized diagnostic tests, Goswami et al. used EBV proteins expressed in human cells to assess the virus-specific IgG response (Goswami et al., 2017). Practicability and sensitivity of this approach were demonstrated by the verification of known immunodominant, and the identification of novel EBV antigens. However, only sixty-two of all EBV genes could be expressed to detectable levels in HEK293 cells. Moreover, probing cell lysates of transfected cells may cause detection of self-proteins by autoantibodies, which may explain the recognition of EBV proteins by sera from EBV-negative donors.

Here, we report on the development of a multiplex dot blot assay (MDB) comprising all annotated open reading frames of EBV expressed in and purified from human cells and describe its validation.

## Methods

### Serum samples and antibodies

Serum samples (n=200) were obtained under institutional review board-approved protocol (project Nr. 112/14) from EBV-seropositive (EBV+) and EBV-seronegative (EBV-) donors based on the results of clinical tests performed in the Institute of Virology, Technical University Munich (ELISA ARCHITECT™ (Abbott) and *recomLine* Immunoblot (Mikrogen)). A donor was considered negative only if no anti-EBNA1-IgG or IgM, anti-VCA-IgG or IgM, or anti-EA-IgM antibodies were detectable. EBV seroconversion panels (SCP) of two donors were bought from DiaMex. All serum samples were stored at -20 °C until use. The murine monoclonal antibody 3D5 directed against the C-terminal His_6_-tag was provided by the monoclonal antibody facility of the Helmholtz Center Munich. Mouse antibodies specific to BZLF1 and BMRF1 were purchased from Argene and Abcam, respectively. Fluorescence-labelled anti-mouse IgG (LI-COR^®^ IRDye 680) and anti-human immunoglobulin isotype (IgM, IgG, IgA)-specific antibodies (LI-COR^®^ IRDye 800) were aliquoted and stored at -20 °C.

### Antigenic proteins

A common expression plasmid (pcDNA3, Invitrogen) was used to clone all 83 annotated open reading frames of the EBV type 1 prototype strain B95.8 (GenBank: V01555.2) as either wildtype or codon-optimized expression constructs. The ORFs of LF1, LF2 and LF3, not present on the B95.8 genome, were derived from the Raji EBV genome (M35547.1). The list of all 86 EBV proteins included is shown in **Table 1**. GFP and the human IgM, IgG1, and IgA constant regions (GenBank: P01871, P01857.1, P01876) were cloned in the same way. By design of the expression plasmids, all open reading frames were expressed as C-terminally His_6_-tagged proteins in HEK293T cells as described (Adhikary et al., 2007). Recombinant proteins were purified from transfected cell lysates using a 8 M urea lysis buffer and Ni-NTA agarose (Qiagen) affinity purification, as described previously, and the proteins detected using the anti-His_6_ mouse monoclonal IgG antibody 3D5 (Adhikary et al., 2007). Due to low expression of the large tegument protein BPLF1, the open reading frame was divided into three partially overlapping fragments (AA1-1535, AA1262-2330, AA2310-3149) and expressed separately. To prevent autocatalytic cleavage, a Ser-to-Gln substitution at amino acid 116 was introduced in BVRF2 (Donaghy and Jupp, 1995). A shorter version of BHLF1 containing all unique sequences but only six instead of eleven NotI repeats was used. In the case of LF3, an expression construct containing all unique sequences plus two instead of 23 SacI repeats was used. Of note, both constructs code for all polypeptide sequences present in the original ORFs. Quantity and size of all recombinant proteins were analyzed by Western blot. Concentrations of proteins were estimated by staining polyacrylamide gels with Coomassie dye and comparing band intensities with those of known concentrations of bovine serum albumin (BSA). All preparations were adjusted to approximately 10 µg/ml. Tetanus/diphtheria vaccine (Td-pur^®^ Astro Pharma) was diluted in 8 M urea buffer (Nückel et al., 2022) and included as positive control.

**Table 1:** EBV proteins included in this analysis.

A73	BALF0/1	BALF1	BALF2	BALF3	BALF4	BALF5	BARF0	BaRF1	BARF1
BBLF1	BBLF2/3	BBLF4	BBRF1	BBRF2	BBRF3	BcLF1	BcRF1	BCRF1	BDLF1
BDLF2	BDLF3.5	BDLF3	BDLF4	BdRF1	BFLF1	BFLF2	BFRF1a	BFRF1	BFRF2
BFRF3	BGLF1	BGLF2	BGLF3.5	BGLF3	BGLF4	BGLF5	BGRF1/BDRF1	BHLF1	BHRF1
BILF1	BILF2	BKRF2	BKRF3	BKRF4	BLLF1	BLLF2	BLLF3	BLRF1	BLRF2
BMLF1	BMRF1	BMRF2	BNLF2a	BNLF2b	BNRF1	BOLF1	BORF1	BORF2	BPLF1
BRLF1	BRRF1	BRRF2	BSLF1	BSLF2/BMLF1	BSRF1	BTRF1	BVLF1	BVRF1	BVRF2
BXLF1	BXLF2	BXRF1	BZLF1	BZLF2	EBNA1	EBNA2	EBNA3A	EBNA3B	EBNA3C
EBNA-LP	LF1	LF2	LF3	LMP1	LMP2A				

### Multiplex dot-blot assay and quantification of measurement

Each concentration-adjusted recombinant protein was spotted on a nitrocellulose membrane. After drying, the membrane was blocked with 5% milk powder in PBS and co-incubated overnight at 4 °C with an anti-His_6_ antibody (clone 3D5) and human serum samples in a 3% milk powder in PBS. The membranes were subsequently washed and incubated with fluorescence-labelled anti-mouse IgG antibody (LI-COR^®^ IRDye 680) and anti-human Ig isotype-specific antibodies (LI-COR^®^, IRDye 800; Biomol, DyLight 800), all at 1:10,000 dilution. The membranes were scanned in a near infrared imaging system (Odyssey FC, LI-COR^®^) that reports results as arbitrary fluorescence units (AFU), returning a CW700 and a CW800 reading for each dot on the membrane corresponding to the protein concentration (anti-His_6_) and the human serum response, respectively. Depending on the Ig subtype analyzed, a standard curve of recombinant His_6_-tagged human IgM, IgG1 or IgA constant region as well as solvent (8 M urea buffer) and Ni-NTA agarose-affinity enriched mock-transfected HEK293T cell lysate were used for specific standardization and background correction, enabling blot to blot comparability.

### Data processing

Given that the AFU values of the CW800 channel are approximately one order of magnitude lower than those of the CW700 channel, all raw CW800 measurements were scaled by a factor of 10 to enhance clarity in graphical representations. Autofluorescence signals caused by the nitrocellulose membrane or the solvent as well as any possible fluorescence due to serum responses against HEK293T proteins were considered background and subtracted from readings for antigenic proteins. Background-subtracted AFU values for proteins in the CW800 and CW700 channels were converted to normalized arbitrary values using a standard curve generated from eight serial dilutions of recombinant His_6_-tagged immunoglobulin constant regions (IgM, IgG1, and IgA Fc) at known concentrations. Fluorescently labeled anti-human isotype-specific secondary antibodies bind directly to these Fc regions, while anti-mouse IgG secondary antibodies bind indirectly via a mouse monoclonal antibody targeting the His-tag. Standard curves were generated from these measurements using statistical software, and samples quantified by interpolation.

Next, the quotient of normalized CW800 and CW700 values was formed to compensate for potential differences in the amount of sample protein spotted on the membrane. This normalized AFU (nAFU) value is used to describe serum responses against viral proteins.

To identify positive antibody responses to the candidate antigens, a protein-specific cutoff was established for each analyzed protein. For IgG and IgA, the cutoff was defined as the mean plus two standard deviations, while for IgM it was defined as the mean plus 2.5 standard deviations. These values were calculated from identical dot blot assays performed with sera from 30 EBV-negative donors. Using this approach, negative control samples were correctly classified with a specificity ranging from 95.2% to 100.0% (mean: 98.1%).

### Software and statistical methods

The distribution of quantitative data is presented by mean, range, and standard deviation. Qualitative data is described by absolute and relative frequencies. Correlations between two parameters were evaluated using Spearman’s correlation coefficient (r_s_). r_s_ values between 1.0 and 0.7, 0.7 and 0.5, 0.5 and 0.3, and below 0.3 were considered very strong, strong, moderate, and low correlations, respectively (Mukaka, 2012). Group differences were assessed by Mann-Whitney U tests. Hypothesis testing was performed at exploratory two-sided 5% significance levels. Data was collected in Microsoft Excel sheets, analyzed using GraphPad Prism 9 and figures finalized in Adobe Illustrator CS.

## Results

### Establishing a multiplex assay to measure antibody responses against the EBV proteome

**Figure 1:**
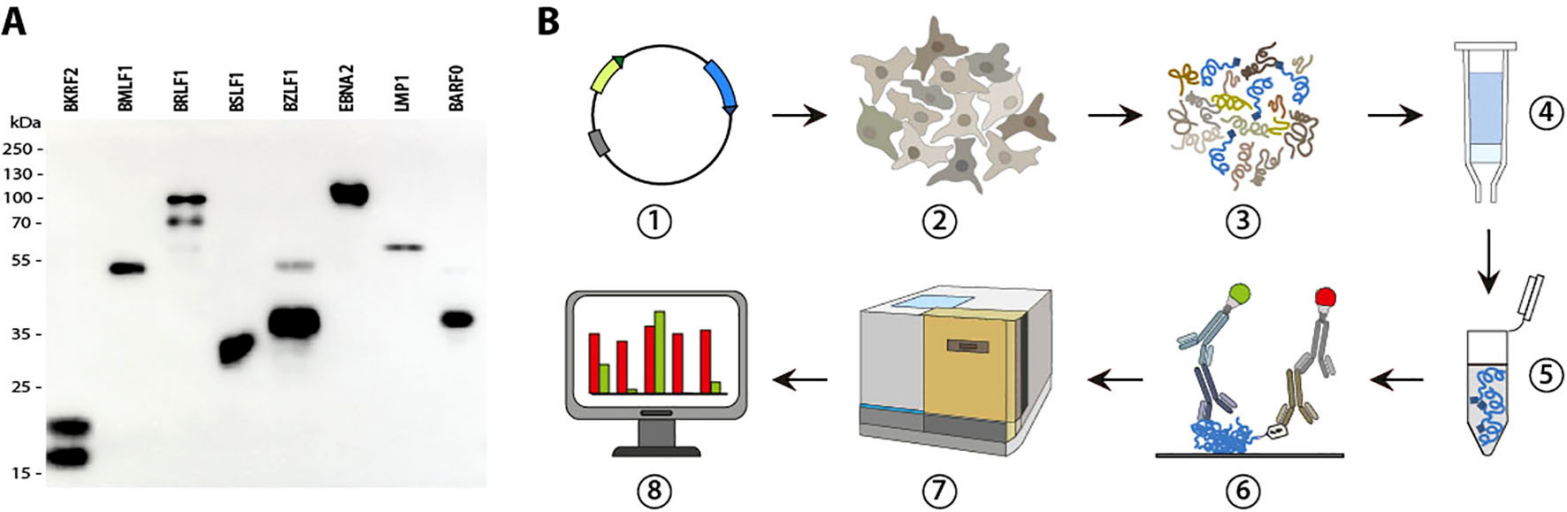
Schematic outline of the multiplex assay. **(A)** Exemplary Western blot of recombinant EBV proteins expressed in HEK293T cells, purified via their C-terminal His_6_-tag, and detected using a His_6_-tag-specific antibody. **(B)** Schematic outline of the multiplex assay: ① Expression vectors for C-terminally His_6_-tagged EBV proteins were constructed and individually transfected in HEK293T cells ②. After four days, the cells were harvested and lysed ③, the lysate loaded on Ni^2+^-NTA columns ④, and the His_6_-tagged proteins eluted with Imidazole ⑤. After verification of integrity and identity, the concentration of proteins was adjusted to approximately 10 µg/ml and 5 µl of each protein spotted on a nitrocellulose membrane and incubated with 1:500 to 1:1000 diluted sera as well as a mouse monoclonal antibody directed against the His_6_-tag ⑥. Bound human and mouse antibodies were detected using IRDye-680RD and IRDye-800CW secondary antibodies, respectively, in a near-infrared detection system ⑦. Antibody responses were quantified by normalizing “AFU, arbitrary fluorescence units” against the amount of spotted protein and a standard ⑧.

The full-length open reading frames of 86 type 1 EBV genes were C-terminally fused with a His-tag, expressed in HEK293T cells and purified over nickel-NTA columns as described (Adhikary et al., 2007). Lysates from GFP or mock transfected HEK293T cells were purified under identical conditions and included as negative controls. Identity, purity, and concentration of the recombinant proteins were analyzed by Western blot using the anti-His_6_ antibody 3D5 (**Figure 1A**).

To profile antibody responses across the entire EBV proteome, purified proteins were immobilized on nitrocellulose membranes and incubated with human sera, as well as with the monoclonal mouse antibody 3D5 targeting the C-terminal His_6_-tag. Bound antibodies were detected using species-specific secondary antibodies conjugated to distinct infrared fluorescent dyes. Signal intensities were quantified with a near-infrared imaging system (Odyssey Fc Imaging System, Li-Cor), which, unlike chemiluminescence-based detection methods, provides a linear dynamic range spanning at least 4 orders of magnitude, thereby eliminating the need for serial serum dilutions.

**Figure 2:**
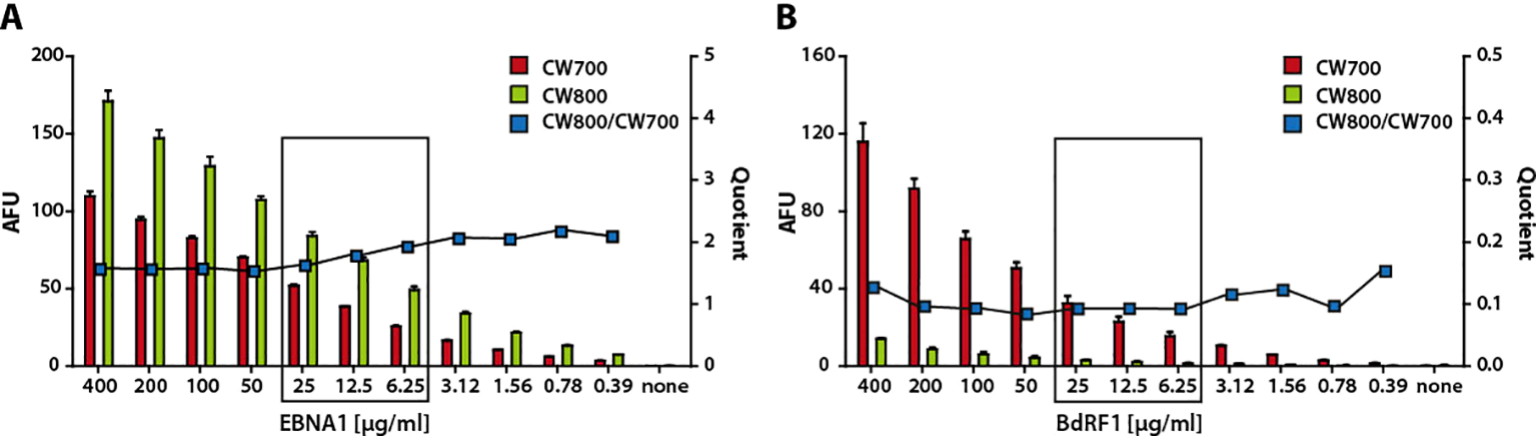
Direct proportionality between the IgG antibody response and protein amount over a wide concentration range. Decreasing amounts of the EBV proteins EBNA1 **(A)** and BdRF1 **(B)** were spotted in triplicate onto membranes, incubated with serum from an EBV-positive donor and with an anti-His_6_ antibody, and analyzed for IgG responses by MDB. Shown are the quantified protein levels (CW 700) and corresponding serum responses (CW 800), both expressed in AFU, arbitrary fluorescence units as mean ± SD. In addition, the ratio of serum response to protein amount (quotient CW800/CW700) is presented. The highlighted rectangle indicates the range of antigen concentrations selected for subsequent experiments.

To define optimal serum dilutions and protein concentrations, recombinant proteins were spotted at varying amounts and probed with sera diluted from 1:10 to 1:10,000. Analysis of 15 serum samples with known antibody titers against EBNA1 and VCA revealed that dilutions of 1:1000 for IgG and IgA, and 1:500 for IgM, yielded optimal signal-to-noise ratios (data not shown). To assess whether differences in protein concentration or pipetting accuracy could bias antigen-to-antigen comparisons, serial protein dilutions were incubated with sera from EBV-positive donors. As illustrated for one donor with high responses to EBNA1 and low responses to BdRF1, antibody reactivity scaled proportionally with the amount of protein spotted (**Figure 2**). Notably, the ratio of antibody response to protein amount remained constant over three orders of magnitude. Thus, normalizing serum responses to the amount of protein spotted enables robust comparisons of antibody reactivity across antigens. Based on these findings, a protein concentration of approximately 50 ng per spot was selected for all subsequent experiments and the ratio of antibody signal to protein concentration used as a proxy for antibody titer.

To enable intermembrane comparisons, internal standards were introduced. The Fc regions of IgM, IgG1, and IgA were expressed as His_6_-tagged proteins in HEK293T cells and applied to the membranes in serial dilutions. Spotted proteins were detected by the anti-His_6_ antibody and by direct binding of the isotype-specific anti-human Ig secondary antibodies. When membranes were spotted with proteins at 50 ng, 100 ng, or 200 ng concentrations and serum responses measured, intermembrane variability in antigen-specific signals decreased from over 400% to less than 20% when normalized to the standards (data not shown).

### Impact of the His_6_-tag on antibody recognition

To assess whether binding of the C-terminal His_6_-specific antibody interferes with recognition of proteins by serum antibodies, particularly at C-terminal epitopes, experiments were performed using the EBV proteins BFRF3, BMRF1, and BZLF1. The C-terminal region of BFRF3 (amino acids 110–176) contains a well-characterized, virus-specific immunodominant domain comprising multiple short peptide epitopes (Fachiroh et al., 2006; Van Grunsven et al., 1994). Likewise, most antibodies against BMRF1 recognize epitopes in its C-terminus (amino acids 300–380) (Middeldorp, 2015) making both proteins suitable for evaluating potential interference from C-terminal tagging. BZLF1, which lacks immunodominant epitopes in its C-terminal region (Tedeschi et al., 1995), served as a control.

**Figure 3:**
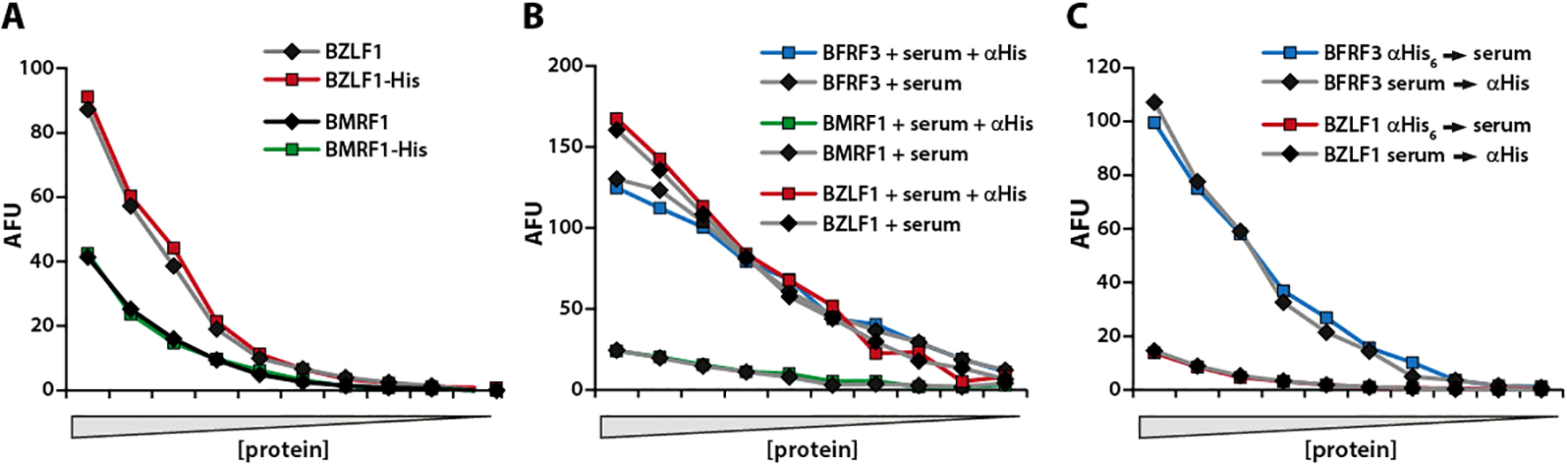
The His_6_-tag does not interfere with serum antibody binding. **(A)** BZLF1 and BMRF1 were expressed in HEK293T cells either with or without a C-terminal His_6_-tag. Decreasing amounts of viral protein content-adjusted lysates were spotted on membranes and IgG responses quantified. **(B)** Identical membranes were spotted with decreasing amounts of the His_6_-tagged EBV proteins BFRF3, BMRF1, and BZLF1, and then incubated either with serum (1:1000) alone, or with serum and the αHis_6_ antibody 3D6. **(C)** Identical membranes were hybridized with the αHis_6_ antibody for one hour, followed by serum for another hour (αHis_6_ serum), or in the reverse order (serum αHis_6_). Serum responses were measured as AFU, arbitrary fluorescent units. Representative results from experiments with serum from three EBV-positive donors are depicted.

To test for interference, BZLF1 and BMRF1 were expressed in HEK293T cells either with or without a C-terminal His_6_-tag, and protein levels in the cell lysates were normalized via Western blot using protein-specific antibodies. Serially diluted lysates were spotted onto membranes, and serum IgG responses were quantified (**Figure 3A**). In a second set of experiments, membranes were spotted with serial dilutions of BFRF3, BMRF1, and BZLF1 and incubated either with serum alone or with a combination of serum and the anti-His_6_ antibody (**Figure 3B**). Alternatively, membranes were incubated sequentially with anti-His_6_ antibody followed by serum, or vice versa (**Figure 3C**). Across all protein concentrations tested, none of these experiments indicated interference from the His_6_-tag, either alone or in complex with its specific antibody.

### Ruggedness of the multiplex assay

**Figure 4:**
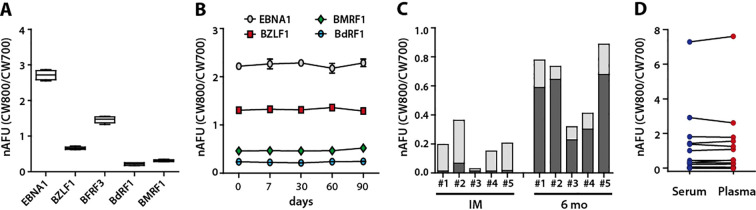
Robustness and versatility of the MDB assay. **(A)** Five different preparations of the indicated five EBV proteins were probed with serum from an EBV-positive donor. Box plots depict the mean, minimum and maximum of the normalized antibody responses (CV: 5-17%, mean 8,5%). **(B)** Identical membranes spotted with EBV proteins were either immediately hybridized with serum from EBV-positive donors or stored at room temperature for the indicated time periods before hybridization. IgG responses to the specified EBV antigens were subsequently measured. (CV: 2.0-5,4%). **(C)** Pairs of identical membranes were hybridized with sera from five patients with IM, infectious mononucleosis, collected during acute illness (IM) and six months (6 mo.) later. Control membranes were processed according to the standard protocol. In parallel, membranes were treated with 6 M urea buffer for 5 minutes after serum incubation, then processed identically to the controls. Columns represent total serum IgG responses to VCA p18, with the dark gray segments indicating the portion of the response remaining after urea treatment. **(D)** Identical membranes were incubated with serum or plasma of the same EBV-positive donors and antibody responses analyzed. A representative example of one donor is shown, with antibody responses against the same antigen connected by a line. Antibody responses are expressed as nAFU. Welch’s t-test: p = 0.9868.

The impact of different antigen preparations on the serum response was assessed by measuring antibody responses against five individual antigen lots from EBNA1, BFRF3 and BZLF1 each in sera from five EBV-positive donors. The mean of the normalized values was then calculated for each antigen lot and percent difference calculated, ranging from −10.11% to 12.31% and thus well within the accepted coefficient of variation (CV) (**Figure 4A**).

Serological screenings, which often involve varying sample numbers and irregular timing, benefit from the ability to prepare antigen-spotted membranes in advance. To assess the functional stability of membrane-bound proteins over time and thus determine the impact of storage duration on assay reproducibility, identical membranes were prepared and either immediately incubated with serum or stored dry at room temperature for up to three months before use with the same sera. As shown in **Figure 4B**, storage did not affect assay reproducibility. IgG responses to four representative antigens remained stable throughout the testing period, demonstrating that pre-prepared membranes retain their functional integrity even after extended storage.

In clinical diagnostics, assessing the avidity of anti-VCA IgG responses can help distinguish acute from past EBV infections in individuals who lack detectable anti-EBNA1 IgG responses (Andersson et al., 1994). The latter group includes rare cases where individuals either fail to develop measurable anti-EBNA1 antibodies or have lost these responses due to immunosuppression (Miller et al., 1987; Henle and Henle, 1981). Treatment with chaotropic agents such as urea, which disrupts weak antibody-antigen interactions, enables differentiation between low-avidity IgG, characteristic of acute infections, and high-avidity IgG, typically found in long-term carriers (Bauer, 2001; Pottgiesser et al., 2012).

To evaluate whether the multiplex dot blot assay could measure antibody avidity, membranes were incubated in parallel with sera from five patients with infectious mononucleosis (IM) during the symptomatic phase and six months post convalescence. Control membranes were processed under standard conditions, whereas test membranes were treated with 6 M urea in PBS for 5 minutes after serum incubation and then processed following the standard protocol. The antibody avidity index (AI) was calculated as the titer after urea treatment divided by the titer of the untreated control. Low-avidity responses were defined as AI < 0.5, and high-avidity responses as AI > 0.6 (Correa et al., 2021; Andersson et al., 1994). As shown in **Figures 4C**, anti-VCA p18 antibodies exhibited low avidity during acute infection and high avidity six months later, demonstrating that the multiplex dot blot assay is suitable for antibody avidity measurements in EBV diagnostics.

To test whether the MDB is suitable for analyzing serum or plasma samples, identical membranes were incubated with serum or plasma of five EBV positive donors and antibody responses measured. As illustrated for one representative donor, no statistically significant differences were observed, indicating that the MDB can reliably process both serum and plasma samples (**Figure 4D**).

### Analytical specificity

To evaluate potential antigenic interference, assays were conducted using sera from 15 EBV-positive donors. Antibody responses to individual EBV antigens were compared with responses obtained when all antigens were tested in combination. As shown in **Figure 4A** for BFRF3, BZLF1, and EBNA1, a very high correlation (R² = 0.98–0.99) was observed between responses to individual antigens and those measured using the multiplex format. Similarly high correlations were also observed for less immunodominant antigens (n>30), which elicited antibody responses in lower percentages of individuals, such as BALF2, BALF4, BLLF1, BLRF2, BMRF1, BZLF2, and the EBNA3 family (data not shown).

To further assess the analytical specificity of the assay, competition experiments were performed using homologous and heterologous antigens. Serum samples from three EBV-positive donors were spiked with a tenfold excess of either homologous or heterologous antigen. Only the homologous antigen led to a marked reduction in antibody binding, confirming the analytical specificity of the assay (**Figure 4B**).

### Assay performance characteristics

To assess the accuracy of the MDB assay, fifteen sera were tested at least five times by three operators. Intra-assay variation for individual analytes within a membrane varied between 4% and 7%, and the intra-assay variation between membranes varied between 7% to 14%. The mean CV for the inter-assay variation for one operator and for three operators ranged from 11% to 16%, and 12% to 19%, respectively, for the different analytes, indicating high reproducibility of the assay (**Table 2**). When membranes with all EBV proteins were compared, variation was 2-19% (intra-assay), 7-23% (inter-assay), and 5-27% (between operators).

**Table 2:** Reproducibility of the multiplex assay: intra-assay variation and inter-assay variation represented as the mean percentage of coefficient of variation (CV).

Analyte	Intra-assay variation (within membrane)	Intra-assay variation (between membranes)	Inter-assay variation (between assays)	Inter-assay variation (between operators)
	mean % CV (n)	mean % CV (n)	mean % CV (n)	mean % CV (n)
Single EBV proteins	6 (24)	10 (48)	14 (48)	16 (96)
EBV protein array	n/a	13 (5)	16 (5)	21 (10)

### Assay specificity and sensitivity

Sera from 30 EBV-negative donors were used and background responses against every protein determined. For each protein, a cutoff was defined as mean responses of all EBV-negative donors plus 2 (for IgG and IgA) and 2.5 (for IgM) standard deviations. When these cut-off values were reapplied to the EBV-negative control, specificity ranged from 95.2% to 100.0% (mean: 98.1%).

To assess sensitivity, sera from healthy donors with unknown EBV status (n=20) were analyzed and results compared to standard clinical tests. In both analyzes identical results were obtained with 18 being EBV-seropositive and two EBV-seronegative (data not shown). When compared with results from the *recomLine* EBV assay, which includes six EBV antigens, concordance for each antigen was nearly 100%. The few discrepancies observed were limited to ambiguous results in the *recomLine* assay (data not shown).

**Figure 5:**
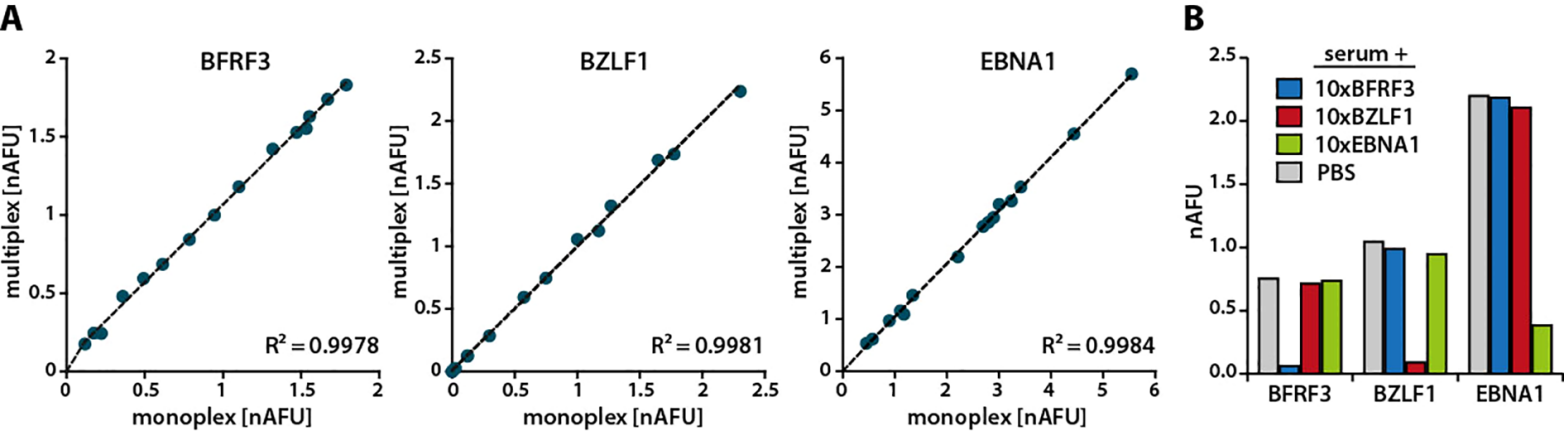
Assessment of the analytical specificity of the MDB assay. **(A)** Dot blot assays were performed using either individual EBV antigens or a multiplex format including all other EBV proteins. IgG responses were measured in sera from 15 EBV-seropositive donors (represented as dots) and expressed as nAFU. Correlations between responses were then analyzed. **(B)** Sera were spiked with a tenfold excess of BFRF3, BZLF1, EBNA1, or PBS as negative control, prior to measuring antibody responses against the respective antigens in dot blot assays. A representative result from one of three donors is shown.

Analysis of sera from IM patients, collected during acute infection (T_0_) and six months later (T_6_), showed largely consistent IgG responses against EBNA1 and VCAp18 across ELISA, immunoblot (recomLine), and MDB methods (**Figure 5**). The only notable difference was that VCAp18 responses at T_0_ were significantly lower in the recomLine assay compared to the other two methods. This is most likely explained by the use of an N-terminally truncated version of VCAp18 in the recomLine test. Antibodies against the N-terminal region emerge early after infection, whereas antibodies to the C-terminal region typically appear several weeks later (Middeldorp, 2015; Bauer, 2001).

Because no internationally accepted reference sera for EBV antigens are available, we evaluated serum responses to EBNA1 and BFRF3 in seroconversion panels (SCP) from two donors at different time points after EBV infection, for which results from commercial tests were available.

Linear regression and Spearman rank correlation (rs) analyzes were performed to assess correlations between the different test systems. Both seroconversion panels showed very strong (rs > 0.84) and statistically significant (p < 0.05) correlations for BFRF3 and EBNA1, indicating high concordance of the MDB with commercial assays (**Table 3**). For EBNA1 in SCP1, no Spearman correlation coefficient or linear regression could be calculated, as anti-EBNA1 IgG responses typically develop months after primary infection and values in all tests remained below threshold.

**Table 3:** Spearman’s rank correlation (r_s_) between EBV-specific IgG antibody responses in MDB and ELISA (architect and liaison).

	Liaison	Multiplex dot blot (MDB)
BFRF3 in SCP1 (n=6)		
Architect	0,892*	0,892*
Liaison		1***
BFRF3 in SCP2 (n=7)		
Architect	0,922**	0,874**
Liaison		0,903**
EBNA1 in SCP2 (n=7)		
Architect	0,957**	0,957**
Liaison		1***

SCP, seroconversion panel; n, number of sera analyzed; two-tailed t-test, * p<0.05, ** p<0.01, *** p<0.00.1.

**Figure 6:**
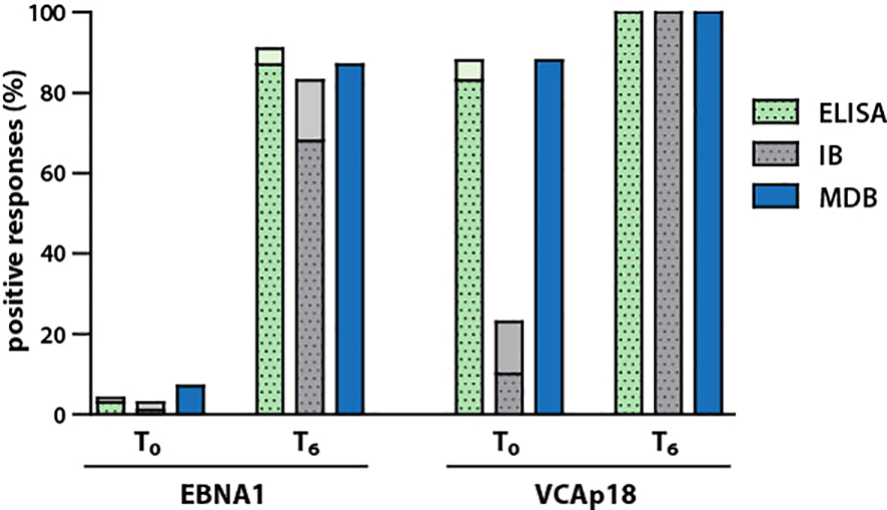
Comparison of IgG responses to EBNA1 and VCA in IM patients using MDB and commercial assays. IgG responses to EBNA1 and VCAp18 were assessed in sera from IM patients at the T_0_, time of acute illness and T_6_, six months later using commercial ELISA and IB, immunoblot, and compared with results from MDB. In ELISA and IB, dotted bars indicate positive responses, while solid bars represent borderline responses.

**Figure 7:**
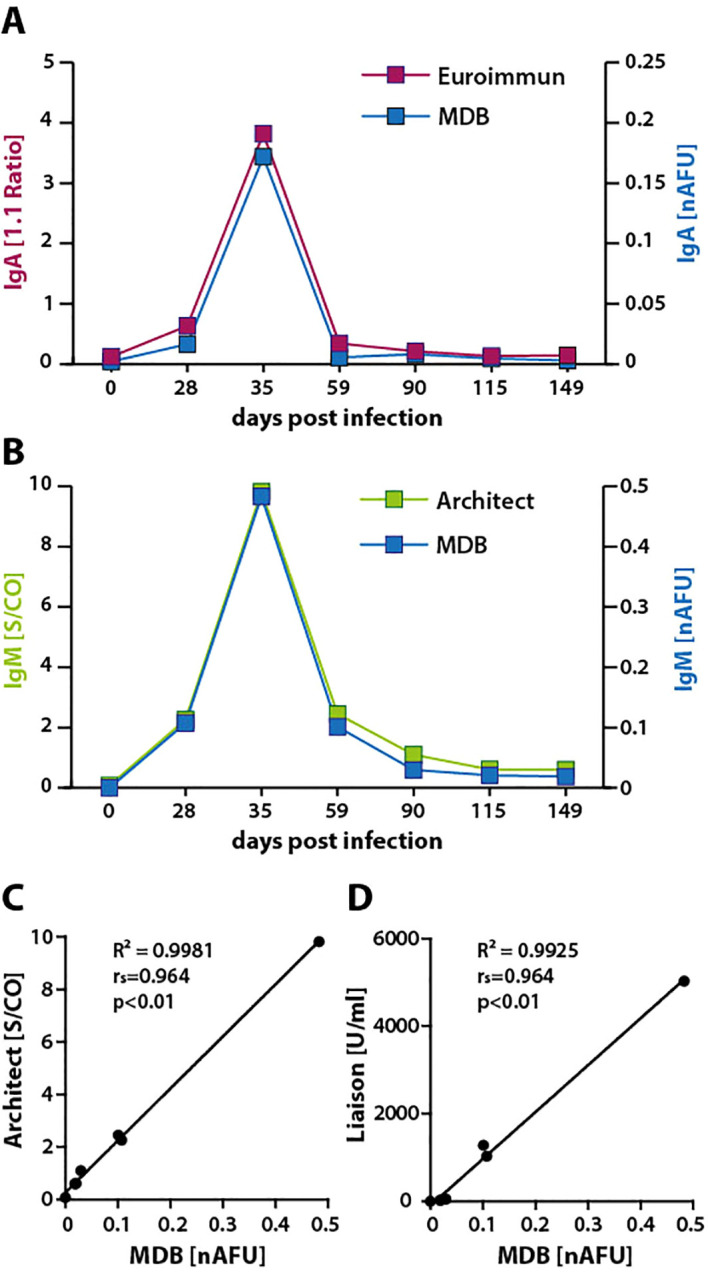
Comparison of IgM and IgA responses to VCAp18 by ELISA and MDB. **(A)** IgA responses to VCAp18 were measured in sera from SCP1 at the indicated time points by ELISA (Euroimmun) and MDB. **(B)** IgM responses to VCAp18 were measured using ELISA (Architect) and MDB. **(C, D)** Correlation of IgM responses obtained by MDB versus ELISA (Architect or Liaison). S/CO, signal-to-cutoff ratio.

Similar comparisons were conducted for IgA and IgM antibody responses against VCAp18 using sera from SCP1 (**Figure 6**). Although direct comparisons of titers were not feasible due to methodological differences, the response dynamics were highly similar. Notably, IgM responses showed very strong correlations between the commercial assays (Architect and Liaison) and the MDB, with r_s_ values approaching 1 (**Figure 7**). These results demonstrate a high degree of concordance between MDB and ELISA measurements, extending to both IgM and IgA responses. .

## Discussion

The measurement of EBV-specific antibody responses serves multiple diagnostic purposes, including the detection of seroconversion, distinguishing acute from past infections, monitoring viral reactivation, and evaluating vaccine efficacy (Niller and Bauer, 2017; Mautner and Middeldorp, 2025). However, clinical utility of currently available commercial assays, most of which target only a few EBV antigens, remains limited for diagnosing most EBV-associated chronic and malignant disorders (Goswami et al., 2017; Hinderer et al., 1999; Rowe et al., 1988; Xiao et al., 2009). As a result, recent research has shifted towards comprehensive profiling of antibody responses across the entire EBV proteome, using recombinant proteins and synthetic (poly)peptides as antigens that are produced via chemical synthesis or through prokaryotic and eukaryotic expression systems (Coghill et al., 2018; Dreyfus et al., 2018; Goswami et al., 2017; Li et al., 2023; Loebel et al., 2017; Schlemm et al., 2016; Xu et al., 2015; Zheng et al., 2011). Despite the identification of several promising antigen candidates, some now in clinical evaluation, significant variability was noted across studies in both the number and identity of EBV proteins targeted by antibodies (Goswami et al., 2017; Liu et al., 2018; Zheng et al., 2011).

Such discrepancies may arise from differences in assay sensitivity and specificity, as well as from variations in antigen preparation affecting protein purity, conformation, and post-translational modifications. In addition, residual prokaryotic components in antigen preparations can lead to variable background signals, impairing the definition of reliable cutoff values. While these approaches are valuable for comparing patient cohorts, they are less suited for precise serological profiling at the individual level.

To overcome these challenges and complement existing methodologies, we developed a multiplex immunoassay capable of simultaneously detecting antibodies against the complete repertoire of full-length EBV proteins expressed in HEK293 cells. These cells are routinely used for producing recombinant infectious EBV, ensuring the presence of biologically relevant post-translational modifications (Feederle et al., 2010).

Similar approaches with full-length antigens expressed in HEK293 cells have been employed in previous studies by Goswami et al (Goswami et al., 2017) and Paudel et al (Paudel et al., 2022). In these studies, lysates from cells expressing single EBV proteins were analyzed either directly or after separation by SDS-PAGE. While the latter approach limits high-throughput applicability, Goswami et al. observed that more than half of EBV-seronegative donor sera reacted with viral protein preparations, likely due to autoantibodies recognizing host proteins present in the lysates. In our own experiments, we also detected measurable humoral responses to total cell lysates in approximately 10–20% of EBV-negative donors. Such background reactivity can inflate positivity thresholds, masking weaker yet biologically meaningful antibody responses and potentially misrepresenting the true number of viral antigens recognized by an individual.

Therefore, all recombinant proteins in our study were engineered with a His_6_-tag and purified from HEK293T lysates, enabling the definition of narrow and consistent cutoff values for all EBV proteins using a panel of 30 EBV-negative donor sera.

Besides reducing background reactivity, the use of purified proteins allowed for adjusting protein concentrations to a range that yields consistent and reliable measurements. At suboptimal concentrations, small fluctuations can produce large deviations in readouts, whereas within our chosen range, calculated serum responses correlated well with fluorescence signals as well as results from commercial ELISAs performed in parallel. On-membrane measurement of protein concentrations also enables comparison of antibody titers across antigens, overcoming the challenge posed when separate ELISAs are used for different targets. Additional advantages of the MDB over ELISA include a broader linear quantitation range spanning several orders of magnitude, which eliminates the need for repeated testing at different dilutions, as well as a higher sample efficiency. Whereas commercial ELISA kits require approximately 10 μl of serum per antigen, only 30 μl is needed to test the entire EBV proteome. Moreover, antigen requirements are reduced by roughly two orders of magnitude, an important benefit when working with EBV proteins, some of which are expressed at low levels. The consistency of different protein preparations supports reproducible antibody measurements over time, and, as with ELISA, assays can be prepared in advance and stored for at least six months without loss of performance. Furthermore, MDB enables measurement of antibody avidity using chaotropic agents such as urea or ammonium thiocyanate, as well as the detection of IgM, IgA, and IgG responses in plasma or serum with sensitivity and specificity comparable to commercial ELISAs. EBV-specific IgA responses are indicative for NPC, while IgM responses as well as IgG affinity measurement aid in distinguishing primary from persistent infections (Nystad and Myrmel, 2007; Niller and Bauer, 2017; Mautner and Middeldorp, 2025).

Thus, the fluorescent-based MDB is a specific, sensitive, reproducible, sample- and antigen-saving method enabling the simultaneous quantitative detection of antibody responses across the entire EBV proteome and beyond. Unlike other multiplex assays, it consistently produces low and narrowly distributed background signals in sera from EBV-negative donors. This feature allows for the reliable definition of positive responses and provides valuable information for the diagnostic evaluation of individual serum profiles. To fully exploit the high-dimensional datasets generated by this approach, machine learning methods, including unsupervised clustering, can be applied to antibody signature profiling, enabling the identification of distinct serological patterns and the stratification of individuals based on comprehensive EBV-specific immune response signatures.

## Data Availability

The raw data supporting the conclusions of this article will be made available by the authors, without undue reservation.
